# Registration for Clozaril Website (eCPMS) Access for Healthcare Professionals on Chebsey Ward, St George’s Hospital – A Quality Improvement Project

**DOI:** 10.1192/bjo.2025.10398

**Published:** 2025-06-20

**Authors:** Prianna Menezes, Ignacio Agell Argiles

**Affiliations:** St George’s Hospital, Staffordshire, United Kingdom

## Abstract

**Aims:** Clozapine is a critical treatment for patients with treatment-resistant schizophrenia, requiring close monitoring through the Clozaril Patient Monitoring Service (CPMS). Access to the electronic CPMS (eCPMS) is vital for healthcare professionals on Chebsey Ward at St George’s Hospital to ensure safe prescribing, dispensing, and monitoring of clozapine. Delays in obtaining eCPMS access can disrupt patient care and treatment continuity. This Quality Improvement Project (QIP) aimed to streamline the registration process and reduce the time taken for healthcare professionals on the ward to gain access to the platform.

The primary aim of this QIP was to reduce the time required for healthcare professionals, including nurses and doctors on Chebsey Ward, to obtain access to the eCPMS website. The project sought to identify barriers to timely registration and implement strategies to expedite the process, improving workflow efficiency and patient safety.

**Methods:** The project was carried out on Chebsey Ward at St George’s Hospital over a four-month period. Initial testing was conducted to assess the average time taken for healthcare professionals to gain eCPMS access. Root cause analysis identified key delays, including administrative bottlenecks, lack of awareness about the registration process, and incomplete applications. Interventions included the creation of a simplified registration guide specific to the ward and closer collaboration with the CPMS registration team to expedite approvals. A post-intervention cycle was conducted to assess the effectiveness of these measures.

**Results:** The baseline audit revealed that healthcare professionals on Chebsey Ward took an average of seven days to obtain eCPMS access. After implementing the targeted interventions, the post-intervention cycle showed a reduction in registration time to an average of three days, representing a 57% improvement. Staff satisfaction with the registration process increased, and feedback highlighted greater clarity and efficiency in obtaining access.

**Conclusion:** The QIP successfully reduced the time taken for healthcare professionals on Chebsey Ward to gain eCPMS access, improving workflow efficiency and ensuring uninterrupted patient monitoring. The structured approach of process simplification and enhanced collaboration with the CPMS team contributed significantly to the improvements. Future steps include sharing the simplified registration guide to other inpatient wards at St George’s Hospital. This project highlights the importance of targeted interventions in overcoming administrative barriers to enhance patient care and safety.

